# Supplementary material for: The Antioxidant Properties of Lavandula multifida Extract Contribute to Its Beneficial Effects in High-Fat Diet-Induced Obesity in Mice
Source: Antioxidants (Basel). 2023 Mar 29;12(4):832. doi: 10.3390/antiox12040832 (PMC10135096; doi:10.3390/antiox12040832)
Supplement: Supplementary file 1 [file antioxidants-12-00832-s001.zip › antioxidants-2288969-supplementary.pdf]

Table S1. RT-qPCR primers sequences.

Gene	Organism	Sequence 5′-3′	Annealing T °C	Accesion Number
Gapdh	Mouse	FW: CCATCACCATCTTCCAGGAG<br>RV: CCTGCTTCACCACCTTCTTG	60	NM_001289726.1
Adipoq	Mouse	FW: GATGGCAGAGATGGCACTCC<br>RV: CTTGCCAGTGCTGCCGTCAT	52	NC_000082.7
Ampk	Mouse	FW: GACTTCCTTCACAGCCTCATC<br>RV: CGCGCGACTATCAAAGACATACG	60	XM_036159053.1
Glut2	Mouse	FW: TCAGAAGACAAGATCACCGGA<br>RV: GCTGGTGTGACTGTAAGTGGG	59	NM_031197.2
Il1β	Mouse	FW: TGATGAGAATGACCTCTTCT<br>RV: CTTCTTCAAAGATGAAGGAAA	60	NC_000068.8
Il6	Mouse	FW: TAGTCCTTCCTACCCCAATTTCC<br>RV: TTGGTCCTTAGCCACTCCTTCC	60	NM_031168.2
Jnk1	Mouse	FW: GATTTTGGACTGGCGAGGACT<br>RV: TAGCCCATGCCGAGAATGA	60	AY383616.1
Lep	Mouse	FW: TTCACACACGCAGTCGGTAT<br>RV: GCTGGTGAGGACCTGTTGAT	60	NC_000072.7
Lepr	Mouse	FW: GCTATTTTGGGAAGATGT<br>RV: TGCCTGGGCCTCTATCTC	60	NC_000070.7
Muc1	Mouse	FW: GCAGTCCTCAGTGGCACCTC<br>RV: CACCGTGGGCTACTGGAGAG	60	NM_013605.2
Muc2	Mouse	FW: GCAGTCCTCAGTGGCACCTC<br>RV: CACCGTGGGGCTACTGGAGAG	60	NC_000073.7
Muc3	Mouse	FW: CGTGGTCAACTGCGAGAATGG<br>RV: CGGCTCTATCTCTACGCTCTCC	60	NM_005960.1
Ocln	Mouse	FW: ACGGACCCTGACCACTATGA<br>RV: TCAGCAGCAGCCATGTACTC	56	U49185.1
Pparα	Mouse	FW: AGGCTGTAAGGGCTTCTTTCG<br>RV: GGCATTTGTTCCGGTTCTTC	62	NC_000081.7
Pparγ	Mouse	FW: GAAAGCTCGTCCACGTCAG<br>RV: CAGTACAGCCCCGATGACTC	60	NC_000072.7
Tff3	Mouse	FW: CCTGGTTGCTGGGTCCTCTG<br>RV: GCCACGGTTGTTACACTGCTC	60	NC_000083.7
Tjp1	Mouse	FW: GGGGCCTACACTGATCAAGA<br>RV: TGGAGATGAGGCTTCTGCTT	56	NC_000073.7
Tlr4	Mouse	FW: GCCTTTCAGGGAATTAAGCTCC<br>RV: AGATCAACCGATGGACGTGTAA	60	NM_021297.3
Tnfα	Mouse	FW: AACTAGTGGTGCCAGCCGAT<br>RV: CTTCACAGAGCAATGACTCC	60	NM_001278601.1
